# Insights into Pediatric Sleep Disordered Breathing: Exploring Risk Factors, Surgical Interventions, and Physical and Scholastic Performance at Follow-Up

**DOI:** 10.3390/children11040388

**Published:** 2024-03-24

**Authors:** Marco Zaffanello, Angelo Pietrobelli, Leonardo Zoccante, Luca Sacchetto, Luana Nosetti, Michele Piazza, Giorgio Piacentini

**Affiliations:** 1 Department of Surgical Sciences, Dentistry, Gynecology and Pediatrics, University of Verona, 37129 Verona, Italy; angelo.pietrobelli@univr.it (A.P.); michele.piazza@univr.it (M.P.); giorgio.piacentini@univr.it (G.P.); 2 Child and Adolescent Neuropsychiatry Unit, Maternal-Child Integrated Care Department, Integrated University Hospital Verona, 37126 Verona, Italy; leonardo.zoccante@aulss9.veneto.it; 3 Otolaryngology-Head and Neck Surgery Department, University Hospital of Verona, 37126 Verona, Italy; luca.sacchetto@univr.it; 4 Department of Pediatrics, Pediatric Sleep Disorders Center, F. Del Ponte Hospital, Insubria University, 21100 Varese, Italy; luana.nosetti@uninsubria.it

**Keywords:** adenoidectomy, adenotonsillectomy, breastfeeding, children, quality of life, pediatric quality of life inventory, pediatric sleep questionnaire, quality of life, sleep-disordered breathing

## Abstract

(1) Background: Sleep-disordered breathing represents a growing public health concern, especially among children and adolescents. The main risk factors for pediatric sleep-disordered breathing in school-age children are tonsillar and adenoid hypertrophy. Adenoidectomy, often in combination with tonsillectomy, is the primary treatment modality for pediatric sleep-disordered breathing. This study aims to comprehensively investigate various risk and protective factors in children with sleep-disordered breathing undergoing adenotonsillar or adenoidal surgeries. We also aim to explore the differences in neuropsychological profiles. (2) Methods: This is an observational, retrospective cohort study. We collected information on adenoidectomy or adenotonsillectomy in children referred to our center. We reviewed the clinical history and preoperative visits and collected data through a telephone questionnaire. The Pediatric Sleep Questionnaire (PSQ) and the Pediatric Quality of Life Inventory (PedsQL) screen sleep-disordered breathing and quality of life, respectively. The data were statistically analyzed using SPSS version 22.0 for Windows (SPSS Inc., Chicago, IL, USA). (3) Results: The study involved 138 patients, but only 100 children participated. A higher percentage of patients with sleep-disordered breathing were observed to have mothers who smoked during pregnancy. A smaller proportion of patients with sleep-disordered breathing habitually used a pacifier. A rise in physical score was associated with a reduced PSQ at follow-up (*p* = 0.051). An increase in the overall academic score was related to a decrease in the PSQ at follow-up (*p* < 0.001). A more significant proportion of patients undergoing adenotonsillectomy were observed to have a history of prematurity and cesarean birth. (4) This comprehensive study delves into the intricate interplay of risk and protective factors impacting children with sleep-disordered breathing undergoing adenotonsillectomy and adenoidectomy.

## 1. Introduction

Sleep-disordered breathing (SDB) is a growing concern in public health, particularly among children and adolescents [1,2]. Among its various forms, obstructive sleep apnea (OSA) is the most prevalent in childhood and adults [3]. The literature has documented extensively the association between pediatric OSA and risk factors such as tonsillar and adenoid hypertrophy and obesity [4]. Additional risk factors associated with pediatric OSA include a family history of snoring, allergic rhinitis, asthma, preterm birth, parental smoking, habitual loud snoring, ethnicity, and certain genetic conditions like Down syndrome or neuromuscular diseases [5,6].

Pediatric SDB, particularly OSA, is linked to a spectrum of complications, ranging from growth deficits to neurocognitive impairments, metabolic dysregulations, cardiovascular issues, and reduced quality of life (QoL) [7,8,9,10,11,12,13].

The recurrence of otitis media (catarrhal or serous type) occurs in cases of adenoid hypertrophy. Recurrent otitis can also refer to recurrent external otitis [4].

Adenoidectomy, often in combination with tonsillectomy, is the primary treatment modality for pediatric SDB [14]. Nonetheless, questions persist regarding the optimal management approach, with some studies suggesting potential benefits from combined adenoidectomy and antibiotic therapy [8,15,16,17]. While surgical intervention, as recommended by prominent medical societies [15,18], offers significant relief for many patients, challenges remain, as not all individuals experience resolution of their symptoms post-operatively [19].

In clinical practice, using tools like the Pediatric Sleep Questionnaire (PSQ) and diagnostic methods such as full overnight polysomnography (PSG) play key roles in identifying and assessing pediatric SDB [20,21,22]. Additionally, evaluating the impact of surgery on the quality of life (QoL) using tools like the Pediatric Quality of Life Inventory (PedsQL) can contribute to optimizing patient care [23].

This study aims to comprehensively and thoroughly investigate a wide range of risk and protective factors in children with SDB undergoing adenotonsillar or adenoidal surgeries while also analyzing these conditions’ clinical implications and consequences. Additionally, it compares the outcomes of patients with SDB to those without SDB, aiming to fully understand the impact of this syndrome on children’s health and identify any differences in postoperative results.

## 2. Materials and Methods

### 2.1. Study Design

This retrospective observational cohort study involved 138 children aged 2 to 11 years who were initially recruited from the Pediatric Sleep Disorders Outpatient Clinic and underwent tonsillectomy or A&T at the Otorhinolaryngology Unit of the Hospital of Verona in Italy from November 2018 to December 2021.

Inclusion criteria comprised pediatric age, children who underwent an adenoidectomy or an A&T, availability of clinical information obtained from medical records and telephone interviews with parents/caregivers, and unambiguous indication for surgery falling into one of the four main groups: tonsillar and adenoidal hypertrophy, history of recurrent adenoiditis or tonsillitis, history of recurrent otitis media or hearing loss, and evidence of SDB.

Exclusion criteria included children who did not undergo an adenoidectomy or an A&T, unavailability of clinical information from medical records or telephone interviews, lack of consideration of factors related to SDB development and consequences, and lack of clear indication for surgery. Failure to obtain informed consent from parents/caregivers for the scientific use of data was also considered.

Clinical information was collected through two methods: first, by reviewing medical records, followed by telephone interviews with the parents/caregivers of children who had previously undergone an adenoidectomy or an A&T. In our study, we considered a wide range of factors, including predictive elements of SDB development and potential consequences of the condition. We included factors influencing SDB risk, like birth weight, gender, and maternal smoking, as well as factors potentially affected by SDB, while also considering factors that could be affected or modified in the presence of such disorders. The Ethics Committee approved the protocol for clinical studies in the provinces of Verona and Rovigo at the Integrated University Hospital (3766CESC). All parents/caregivers provided written informed consent for the scientific use of the data.

### 2.2. Medical Records

#### 2.2.1. ENT Characteristics before Surgery

In our comprehensive analysis of medical records, historical data, and preoperative visits, we meticulously compiled the following essential information:

1. Historical cases of recurrent tonsillitis, with tonsil classification based on estimation using the Brodsky scale. Tonsillar hypertrophy was explicitly defined as grades III and IV [15].
2. Adenoidal hypertrophy.
3. History of recurrent otitis media and/or hearing loss.
4. Diagnosis of hearing loss, carefully assessed through clinical presentations and audiometric tests. Pathological conditions were identified using type B and C tympanograms.
5. Antibiotic therapy was undertaken as a treatment for recurrent tonsillitis before surgery.
6. Preoperative PSG, if available.

The indication for the surgery was classified into four main groups:

(i) Tonsillar and adenoidal hypertrophy, carefully assessed through comprehensive preoperative physical examinations.
(ii) Documented history of recurrent adenoiditis or tonsillitis.
(iii) History of recurrent otitis media with effusion and hearing loss.
(iv) Evidence of SDB, either instrumental or clinical.

#### 2.2.2. Cardio–Respiratory Polysomnography

During the overnight assessment, cardio–respiratory polysomnography (PSG) was conducted using a portable ambulatory device (SOMNOscreen PSG, developed by SOMNOmedics GmbH in Randersacker, Germany). This advanced device continuously monitored various key physiological parameters relevant to sleep-related studies. These parameters included nasal airflow, thoracic and abdominal respiratory movements (measured using thoracic and abdominal belts), arterial oxygen saturation (SpO_2_), heart rate (measured via a finger probe), electrocardiogram (ECG), body position (measured via a mercury sensor), and tracheal sounds (recorded through a microphone) [10].

The device was affixed to the participant between 9:00 PM and 8:00 AM, capturing data throughout the night while the individual remained comfortably at home. The recorded data underwent manual and automatic evaluations to ensure a comprehensive analysis. The DOMINO software, specifically SOMNOmedics v.2.6.0, was used for automated analysis. Additionally, experienced technicians manually reviewed the data to obtain accurate results. Respiratory events were assessed following the guidelines provided by the American Academy of Sleep Medicine (AASM) [15].

An AHI score of ≥1 event per hour is defined as OSA [24]. Desaturation was considered if there was a minimum 3% decrease in oxygen levels. All oxygen desaturations (SpO_2_) were quantified relative to the baseline SpO_2_ (%) mean and the minimum SpO_2_ (%). The Oxygen Desaturation Index (ODI, events per hour) was determined by dividing the total number of desaturations by the estimated total sleep time per hour. This metric provided additional information about the severity and frequency of oxygen level fluctuations during sleep.

#### 2.2.3. Questionnaire at Follow-up

The telephone questionnaire was divided into three sections: (1) questions about general and perinatal history, (2) administration of the PSQ, and (3) administration of the PedsQL (physical and school domains).

#### 2.2.4. Case History

The first part of the questionnaire consisted of general questions regarding the perinatal period. In particular, the following information was investigated: birth weight and length, gestational age, and pacifier use. Participants were divided into those who did not use a pacifier or used it for less than two years compared to those who maintained the habit longer. Other information included active maternal smoking during pregnancy, atopy, malocclusion, and neuropsychiatric disorders. Information regarding COVID-19 infection (symptomatic or asymptomatic) was also collected. Parameters at follow-up included the child’s weight, height, and BMI.

#### 2.2.5. Pediatric Sleep Questionnaire (PSQ)

The PSQ (Pediatric Sleep Questionnaire) is a valid tool for assessing overall SDB, including primary snoring and OSA. It consists of 22 questions covering various aspects of sleep problems in children, such as snoring frequency, loud snoring, observed apneas, breathing difficulties during sleep, daytime sleepiness, distractible or hyperactive behavior, and other common symptoms associated with pediatric SDB. These questions can be methodically divided into three distinct groups: nighttime symptoms, daytime symptoms, and cognitive symptoms. The PSQ facilitates the identification of potential cases of pediatric SDB through careful assessment of these symptoms.

Each question within the PSQ requires responses in the form of “yes”, “no”, or “do not know”, reflecting the participant’s perception of the child’s sleep-related experiences. Consequently, the total score is calculated by dividing the sum of positive responses (yes) by the total number of reactions, excluding those marked as “do not know”. This standardized approach ensures a fair and accurate representation of the child’s sleep-related problems.

As a result, the derived total score ranges from 0 to 1, with a critical cutoff point of 0.33 used to distinguish patients exhibiting SDB symptoms from those who do not. This established threshold has been validated and endorsed by Chervin et al. [21]. Based on their PSQ scores, we categorized patients into two groups: those with a normal score (PSQ ≤ 0.33; PSQ−) and those with a pathological score (PSQ > 0.33; PSQ+).

The PSQ has garnered considerable recognition for its effectiveness in screening patients suspected of having SDB. Its ability to comprehensively assess various aspects of sleep disorders, combined with the reliability of the calculated total score, confirms its utility as a robust screening tool [25,26,27].

#### 2.2.6. Questionnaire on Quality of Life in Children (PedsQL)

The PedsQL 4.0 Generic Core Scales is a comprehensive questionnaire to assess Health-Related Quality of Life (HRQoL) in children and adolescents aged 2 to 18. This instrument comprises 23 questions divided into four distinct domains. The assessment involves two parallel versions of the questionnaire, one to be completed by the child and the other by the parent or primary caregiver. Each question asks participants to rate the frequency of occurrence of a specific problem over the past month on a scale from 0 to 4. These ratings reflect the perceived impact of various health-related aspects on the child’s life. In particular, we investigated the physical score (PedsQL) and the school score (PedsQL), expressed as percentage scores. This systematic approach ensures a comprehensive and reliable assessment of the child’s HRQoL across the specified domains [28].

It is essential to emphasize that, in cases where more than 50% of responses are missing or not provided by the participant, the overall score cannot be accurately calculated.

### 2.3. Statistical Analysis

The statistical analysis described focuses on exploring differences in the percentage distributions of categorical variables among different groups of patients. The chi-square test (Fisher exact test) was used for categorical variables. The percentages of patients with defined categorical variables within the PSQ− and PSQ+ groups were compared. Additionally, the percentages of patients with defined categorical variables between the adenoidectomy and A&T patient groups were compared.

A statistical analysis of continuous variables in the PSQ− and PSQ+ patient groups was performed. The mean and standard deviation (S.D.) are provided for each group, along with 95% confidence intervals (C.I. 95%) for the mean. We conducted the Kolmogorov–Smirnov test on a sample to ascertain the normality of the continuous data distribution. If the data distribution was non-normal, the Mann–Whitney U test was performed. Furthermore, the result of the Mann–Whitney test, including the associated *p*-value, is reported.

A multiple linear regression analysis was conducted. In this analysis, the dependent variable was the percentage of PSQ score (%) at follow-up, including the independent variables (or predictors). For each independent variable, several parameters were provided: T, indicating the value of the estimated coefficient for each independent variable in the regression; Standard Error, indicating the standard error of the estimated coefficient; Beta, representing the standardized beta coefficient, indicating how much the dependent variable changes in terms of standard deviations when the independent variable increases by one standard deviation; *t*, representing the *t*-value, which is the ratio of the estimated coefficient to the standard error of the coefficient; and *p*-value for each independent variable, i.e., the statistical significance of the variable’s effect on the dependent variable; the 95% C.I. for B, representing the 95% confidence interval for the estimated coefficient of the independent variable.

*p* < 0.05 was considered statistically significant for all tests employed. However, we acknowledge that the *p*-value in our study may have been influenced by factors such as the small sample size, risk of bias, and random error. Therefore, we will also consider statistical significance with a *p*-value between 0.05 and 0.1, providing a reasonable explanation for this value and other evidence supporting the relationship [29].

The data were recorded in a Microsoft^®^ Excel^®^ database for Windows 11 and statistically analyzed using SPSS version 22.0 for Windows (SPSS Inc., Chicago, IL, USA).

## 3. Results

Of the 138 enrolled patients (65% male), 100 participated. Therefore, 38 patients (27.5%) declined to participate in the survey or were not contacted and were thus excluded from the analysis. Among them, peri/postoperative complications were observed in five children who experienced issues such as coughing, bleeding, or incomplete adenoid removal after surgery.

**Figure 1:**
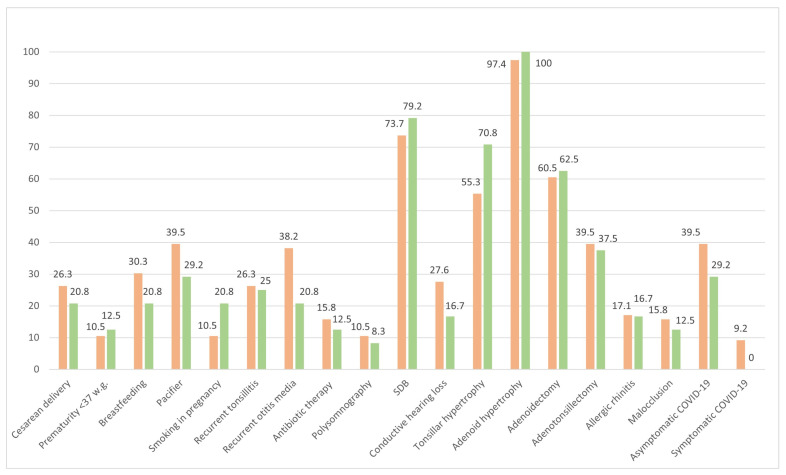
This figure displays the percentage of patients for each categorical variable included in the normal PSQ score (PSQ−; orange bars; *n* = 76) and pathological PSQ score (PSQ+; green bars; *n* = 24) categories. Legend: PSQ, paediatric sleep questionnaire; SDB, sleep-disordered breathing.

**Figure 2:**
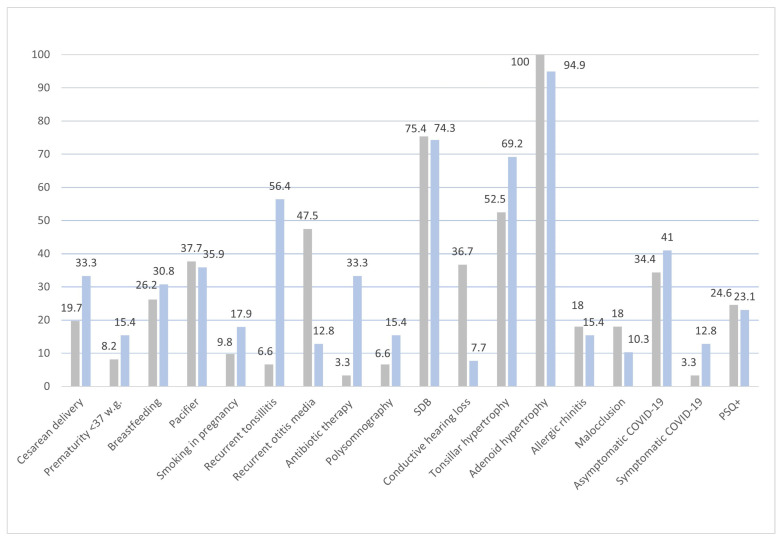
This figure illustrates the percentage of patients for each categorical variable included in the adenoidectomy (grey bars; *n* = 61) and adenotonsillectomy (blue bars; *n* = 39) categories.

Seventy-six patients tested PSQ− while twenty-four tested PSQ+. Among them, sixty-one patients underwent an adenoidectomy, while thirty-nine underwent an A&T. The percentages of patients categorized as PSQ− and PSQ+ for each variable of interest are reported in Figure 1, and the percentages of patients who underwent an adenoidectomy or an A&T for the same variables are shown in Figure 2.

Although not statistically significantly different, postoperative follow-up revealed a lower percentage of children with a history of recurrent otitis media in the PSQ+ group (20.8%) compared to those in the PSQ− group (32.8%), and of hearing loss in the PSQ+ group (16.7%) compared to those in the PSQ− group (27.6%) at follow-up. A higher percentage of patients in the PSQ+ group had mothers who smoked during pregnancy (20.8%) compared to those in the PSQ− group (10.5%). Additionally, a lower proportion of patients in the PSQ+ group had a pacifier use habit (29.2%) than those in the PSQ− group (39.5%). Only a limited percentage had undergone preoperative respiratory polysomnography, and the number of patients did not differ statistically between the two groups. Interestingly, no difference was found in the percentage of children with and without allergic rhinitis between the PSQ+ and PSQ− groups. No child was affected by allergic asthma.

Patients with recurrent tonsillitis were more likely to have undergone an A&T (56.4%) than an adenoidectomy alone (6.6%; *p* < 0.001). Children with recurrent otitis media were more likely to have had an adenoidectomy (47.5%) than an A&T (12.8%; *p* < 0.001). Children with conductive hearing loss were more frequently subjected to an adenoidectomy (36.7%) than to an A&T (7.7%; *p* = 0.005). Children with tonsillar hypertrophy were more likely to have had an A&T (69.2%) than an adenoidectomy alone (52.5%; *p* = 0.002). No difference was found in the percentage of children with and without allergic rhinitis between the adenoidectomy and A&T groups. Antibiotic therapy was more frequently performed in children who underwent an A&T (33.3%) than in those who underwent an adenoidectomy alone (3.3%; *p* = 0.005).

Although not statistically significantly different, premature delivery and cesarean delivery were more common in patients who underwent an A&T (14.4% and 33.3%, respectively) than in those who underwent an adenoidectomy alone (8.2% and 19.7%, respectively). Furthermore, although not statistically significant, the percentage of children who underwent an A&T (15.4%) was higher than those who underwent an adenoidectomy alone (6.6%). No significant differences were observed in the percentage of subjects in the PSQ+ group between those who underwent adenoidectomy and A&T.

The comparison of continuous variables between patients categorized as PSQ− and PSQ+ is reported in Table 1.

No significant differences (*p* ≥ 0.05) were found in birth weight, age at follow-up, and BMI z-score between children who scored PSQ− and those who scored PSQ+. However, physical and school scores (PedsQL) were higher in children with PSQ−, with statistically significant results (*p* < 0.05). Birth weight, age at follow-up, and BMI z-score values may not have been influenced by belonging to the two groups, while physical and school scores (PedsQL) appear to have been influenced by PSQ scores.

Table 2 summarizes the results of a multiple regression analysis examining how various independent variables predict PSQ score (%) at follow-up.

The regression results can be interpreted as follows:

There was a significant (*p* = 0.038) positive association between the age at surgery and the PSQ score (%), indicating that older age at surgery was associated with higher PSQ scores (%) at follow-up. Additionally, a significant (*p* = 0.014) negative association was observed between the age at follow-up and the PSQ scores (%), suggesting that older age at follow-up was associated with lower PSQ scores. Although not statistically significant (*p* = 0.051) at conventional levels, a negative association was noted between physical score (PedsQL) at follow-up and PSQ score (%). Moreover, a highly significant (*p* < 0.0001) negative association was found between the school score (PedsQL) at follow-up and PSQ score (%), indicating that lower school scores were strongly associated with higher PSQ scores at follow-up.

## 4. Discussion

The results of our study demonstrated a lower percentage of children with a history of recurrent otitis media and conductive hearing loss who had undergone an adenoidectomy compared to an A&T. Additionally, children with recurrent tonsillitis were more likely to have undergone an A&T. Despite the different surgical approaches, both A&T and adenoidectomy led to comparable postoperative respiratory outcomes during sleep, as indicated by the absence of significant disparities in pathological PSQ scores (%).

A higher percentage of patients with pathological PSQ scores (%) at follow-up had mothers who smoked during pregnancy compared to those with non-pathological PSQ scores. This aligns with the exposome concept, as environmental exposures during pregnancy can have a significant impact on birth outcomes, and pathways such as sleep disorders, expressed as OSA, often manifest clinically later on [30]. In contrast, fewer patients with pathological PSQ scores (%) had a pacifier-use habit. A history of premature birth and cesarean delivery was more frequent in children who had undergone an A&T. At follow-up, better physical and school performance were mainly associated with normal PSQ scores (%). Specifically, increased physical and school performance was associated with decreased PSQ scores (%).

In examining the complexities of OSA in the young, it is essential to consider various risk factors and comorbidities. Children and adolescents with comorbidities such as obesity, neurological, developmental, or craniofacial abnormalities, asthma, or severe OSAS are at high risk for residual OSA [31]. Additionally, children with otitis media with effusion exhibit significant symptoms of OSAS [32]. At a follow-up appointment, children with recurrent tonsillitis more frequently underwent an A&T rather than an adenoidectomy [18]. Recurrent tonsillitis can serve as an indication for tonsillectomy based on specific criteria outlined by medical guidelines [33]. Finally, a reduced percentage of children with a history of recurrent otitis media and hearing loss had persistent SDB after adenoidectomy surgery [14].

A child’s age plays a crucial role in the development of OSAS. In younger children, there is a greater propensity to exhibit more severe OSAS, presumably due to age-related anatomical and physiological differences [34]. Consequently, it is advisable to monitor younger children closely after A&T [35]. The persistence of OSAS after ENT procedures has been associated with the presence of allergic rhinitis [36,37,38]. In our study, children with SDB were, on average, younger than those without SDB, and no significant differences were found regarding allergic rhinitis between the two groups, whether they underwent an adenoidectomy or an A&T.

Assessing the head and neck area is crucial to determining other causes of upper airway obstruction in addition to tonsils and adenoids. Drug-induced sleep endoscopy (DISE) is essential for diagnosing upper airway obstructions in OSA patients, guiding treatment decisions, and improving surgical outcomes [39]. Evidence suggests a connection between upper airway obstruction and specific patterns of oral breathing stemming from craniofacial development, implying an intertwined interdependence between the two factors [40]. Improper alignment of teeth and jaw, known as malocclusion, can also contribute to SDB by restricting air passage through the nose and throat [41]. However, in our study, no significant differences were found in the percentage of children with malocclusion between those with and without persistent SDB. Malocclusion was associated with specific behavioral habits, such as lack of pacifier use and breastfeeding [42]. In our study, children without SDB more frequently had a history of pacifier use in infancy. However, prolonged pacifier use can negatively affect mouth and teeth development, potentially leading to issues like sleep apnea in children [43]. Despite some evidence, it is impossible to definitively conclude an association between specific malocclusion traits and SDB [44]. Overall, the effects of pacifier use are multifaceted, impacting breastfeeding practices, dental health, SIDS risk, and hospitalization time in preterm infants [43,45,46].

Although breastfeeding has been associated with a lower risk of developing SDB in children [47], we did not observe a significant difference between breastfed and non-breastfed infants for the persistence of SDB. A relationship between tobacco smoke exposure and SDB has been reported in studies. A significant association between secondhand smoke and SDB in children was found [48]. In our study, many children whose mothers smoked during pregnancy exhibited persistent SDB. Ramirez et al. found a significant correlation between prenatal tobacco smoke exposure and mild symptoms of SDB during early childhood in a dose-dependent manner [49]. Tobacco smoke exposure during pregnancy can lead to various adverse perinatal outcomes, such as low birth weight, preterm birth, and perinatal mortality. Additionally, Tan et al. reported that children whose mothers were exposed to smoke during pregnancy had a 2.6-fold increase in their risk of developing OSAS [50]. Rapaport Pasternak et al. have also reported similar results [51].

Pediatric OSAS can have some behavioral consequences during childhood, both externalizing and internalizing, such as difficulties regulating behaviors, aggression, impulsivity, and hyperactivity [52]. Other neuropsychological symptoms previously reported as associated with SDB include internalizing problems such as anxiety, depression, difficulty in controlling emotions [53], attention-deficit/hyperactivity disorder (ADHD), daytime sleepiness, somatization, depression, aggression, and non-compliant social behaviors [15,54,55]. Joosten et al. argue that comorbidities related to OSAS, such as hyperactivity, poor attention, hypertension, growth defects, or enuresis, should raise a physician’s suspicion of the presence of SDB [56]. Consistent with this, we found that poorer physical and school performances were associated with the persistence of SDB. Specifically, a one-point increase in physical and school performance was related to a decrease in average PSQ scores (%). Finally, delaying the age of ENT surgery was associated with increased PSQ scores (%) at follow-up.

The findings of our study could help to identify children at higher risk of persistent SDB and implement appropriate preventive or therapeutic measures. However, our study has some limitations, such as being based on a specific analysis in a single reference center, which may limit the generalizability of our conclusions to all pediatric patients with SDB. Therefore, further research is needed to confirm and expand upon these results.

Another limitation of our study is its observational and retrospective design. Data collected via telephone interviews may be subject to information bias. Finally, when preoperative PSG was unavailable, we recorded symptoms in the medical records and classified them as SDB. Since the number of patients who underwent preoperative PSG was limited, we considered the presence of clinical and instrumental symptoms for diagnosing SDB.

The study’s strengths lie in its comprehensive analysis of various factors related to pediatric SDB and its association with adenoidectomy and A&T. It provides valuable insights into the prevalence of SDB and its persistence post-surgery, shedding light on potential risk factors and outcomes. Additionally, the study’s focus on monitoring health trends over time adds depth to our understanding of SDB incidence and associated factors in the pediatric population.

## 5. Conclusions

In conclusion, this comprehensive study delves into the intricate interplay of risk and protective factors impacting children with SDB undergoing adenoidectomy and A&T. The investigation scrutinizes the clinical ramifications of these conditions and undertakes a comparative analysis between SDB and non-SDB patients, shedding light on the syndrome’s profound implications on pediatric health. Despite the diverse surgical modalities employed, both adenotonsillectomy and adenoidectomy demonstrate comparable postoperative respiratory outcomes during sleep, highlighting the efficacy of these interventions. Furthermore, this study underscores the influence of maternal smoking during pregnancy and pacifier usage habits on postoperative outcomes, alongside identifying associations between surgical procedures and perinatal factors like premature and cesarean deliveries. Notably, superior physical and academic performance post-surgery correlates strongly with normalized PSQ scores. This emphasizes the pivotal role of adequate intervention in mitigating the adverse effects of SDB on children’s well-being.

## Figures and Tables

**Table 1:** This table displays the results of a statistical analysis of continuous variables in two patient groups: those who did not exhibit symptoms of SDB (PSQ−, *n* = 76) and those who did (PSQ+, *n* = 24). The variables’ mean and standard deviation (S.D.) are reported for each group, along with a 95% confidence interval (C.I. 95%) of the mean, indicated by the lower and upper limits. The Mann–Whitney test was conducted to determine whether the two groups had significant differences (*p* < 0.05) for the continuous variables. T_−1_ refers to birth variables, T_0_ refers to variables at surgery, and T_1_ refers to variables at follow-up. Significant *p*-values are highlighted with an asterisk (*) for emphasis.

Continue Variables	PSQ− (*n* = 76)	C.I. 95% (Lower–Upper)	PSQ+ (*n* = 24)	C.I. 95% (Lower–Upper)	Mann–Whitney Test (*p*-Value)
	Mean (S.D.)		Mean (S.D.)		
PSQ (%), T_1_	16.2 (9.0)	−34.3–(−25.7)	46.2 (10.0)	−34.7–(−25.4)	<0.001 *
Birth weight (grams), T_−1_	3249 (575)	−537–(−21)	3507 (677)	−570–53	0.086
Length (cm), T_−1_	50.57 (2.95)	−1.41–1.46	50.54 (3.53)	−1.59–1.64	0.267
Age at intervention (years), T_0_	5.20 (1.6)	−0.70–0.73	5.13 (1.44)	−0.68–0.71	0.904
Age follow-up (years), T_1_	7.13 (1.69)	−0.07–1.47	6.42 (1.54)	−0.05–1.44	0.061
Follow-up (years), T_1_ − T_0_	1.98 (0.87)	0.30–1.07	1.29 (0.70)	0.33–1.03	0.001 *
BMI z-score, T_1_	0.13 (1.56)	−1.28–0.20	0.67 (1.67)	−1.32–0.24	0.086
Physical score (%), T_1_	0.91 (0.09)	0.05–0.18	0.81 (0.23)	0.01–0.21	0.022 *
School score (%), T_1_	0.86 (0.16)	0.08–0.26	0.68 (0.27)	0.05–0.29	0.003 *

**Table 2:** This table shows the results of a multiple regression analysis. A dependent variable, specifically, PSQ score (%) at follow-up, is studied about numerous independent variables. The outputs include estimated coefficients, standard errors, *t*-values, *p*-values, and 95% confidence intervals (95% C.I.) for the coefficients. Additionally, Beta represents the standardized coefficient, indicating how much the dependent variable changes in terms of standard deviation when the independent variable increases by one standard deviation. The *p*-value provides information on the statistical significance of the effect of the independent variables on the dependent variable. T_−1_ refers to birth variables, T_0_ refers to variables at surgery, and T_1_ refers to variables at follow-up. Significant *p*-values are highlighted with an asterisk (*) for emphasis. QoL denotes quality of life.

Variables Entered in the Regression Analysis	Dependent Variable: PSQ Score (%), Follow-Up	T	Standard Error	Beta	*t*-Value	*p*-Value	95% C.I.	
							Lower Limit	Upper Limit
Birth weight, birth length, age at surgery, age at follow-up, BMI z-score at follow-up, physical score (PedsQL), and school score (PedsQL).	Age at surgery (years), T_0_	3.503	1.667	0.337	2.101	0.038 *	0.193	6.812
	Age at follow-up (years), T_1_	−3.811	1.515	−0.404	−2.516	0.014 *	−6.818	−0.804
	Physical score (%), T_1_	−20.892	10.577	−0.195	−1.975	0.051	−41.890	0.105
	School score (%), T_1_	−30.891	7.680	−0.399	−4.022	<0.001 *	−46.138	−15.644

## Data Availability

The datasets presented in this article are not available. The data are not publicly available due to privacy.
